# Identification of Probable Early-Onset Biomarkers for Tuberculosis Disease Progression

**DOI:** 10.1371/journal.pone.0025230

**Published:** 2011-09-23

**Authors:** Jayne S. Sutherland, Philip C. Hill, Ifedayo M. Adetifa, Bouke C. de Jong, Simon Donkor, Simone A. Joosten, Lizet Opmeer, Marielle C. Haks, Tom H. M. Ottenhoff, Richard A. Adegbola, Martin O. C. Ota

**Affiliations:** 1 Vaccinology Theme Group, Medical Research Council Unit, Fajara, The Gambia; 2 Department of Infectious Diseases, Leiden University Medical Center, Leiden, The Netherlands; National AIDS Research Institute, India

## Abstract

Determining what constitutes protective immunity to TB is critical for the development of improved diagnostics and vaccines. The comparison of the immune system between contacts of TB patients, who later develop TB disease (progressors), versus contacts who remain healthy (non-progressors), allows for identification of predictive markers of TB disease. This study provides the first comprehensive analysis of the immune system of progressors and non-progressors using a well-characterised TB case-contact (TBCC) platform in The Gambia, West Africa. 22 progressors and 31 non-progressors were analysed at recruitment, 3 months and 18 months (time to progression: median[IQR] of 507[187–714] days). Immunophenotyping of PBMC, plasma cytokine levels and RT-MLPA analysis of whole blood-derived RNA was performed to capture key immune system parameters. At recruitment, progressors had lower PBMC proportions of CD4+ T cells, NKT cells and B cells relative to non-progressors. Analysis of the plasma showed higher levels of IL-18 in progressors compared to non-progressors and analysis of the RNA showed significantly lower gene expression of Bcl2 but higher CCR7 in progressors compared to non-progressors. This study shows several markers that may predict the onset of active TB at a very early stage after infection. Once these markers have been validated in larger studies, they provide avenues to prospectively identify people at risk of developing TB, a key issue in the testing of new TB vaccines.

## Introduction

Close to one-third of the world's population is infected with *Mycobacterium tuberculosis* (MTb), the causative agent of tuberculosis (TB), with infection rates highest in poverty-stricken countries in Africa and Asia [1]. The majority of infected persons remain asymptomatically (latently) infected with the pathogen, while 10% progress to active TB within their lifetime, resulting in 2 million deaths per year [1]. A better understanding of what constitutes protective immunity to TB is critical for development of improved diagnostics, treatment protocols and vaccines.

The abundance of latently infected individuals world-wide constitutes an extremely large reservoir which fuels TB reactivation and subsequent transmission. However, the relatively low proportion of people that progress to active TB disease suggests that natural immunity to MTb is the general rule, although this also complicates evaluation of intervention studies. The majority of TB biomarker studies to date have focused on differences between subjects with active TB compared to latently infected counterparts [2]–[5]. These have shown the unequivocal role of CD4+ T cells and IFN-γ production in TB immunity [2]–[5], yet do not allow distinction between the underlying cause of progression to active TB and the dynamics of immune changes leading to or resulting from this progression. Other potential immune markers for determining susceptibility or protection to TB, including T cell and B cell subsets [6], type I IFN signalling pathway [7] and apoptotic and innate immune regulators [8] all need to be validated in longitudinal cohort studies that monitor contacts of TB cases (TB case-contact study (TBCC)) for TB disease progression [9]. One such study in The Gambia followed 2348 contacts of TB cases for 2 years resulting in 26 progressors of which half were positive by TST at recruitment and half were positive by ELISPOT [10]. Other studies have examined the predictive values of IFN-γ release assays (IGRA) and TST responses at baseline but have also shown inconclusive results [11], [12]. Clearly, more complex immunological parameters need to be assessed in order to determine more sensitive bio-signatures of protection or susceptibility. This will not only aid in development of effective TB vaccines but will ultimately reduce TB transmission rates by enabling identification and early-treatment of susceptible individuals.

This study provides the first detailed description of the immunological differences between TB progressors and non-progressors at early time points after contact with the index TB case, in most cases many months before the onset of disease. We compared plasma cytokine levels, peripheral blood immune cell phenotypes and whole blood RNA gene expression. These data provide an initial platform for determining biomarkers of protective immunity to TB.

## Materials and Methods

### Ethics statement

This study was conducted according to the principles expressed in the Declaration of Helsinki. Ethical approval was obtained from the Gambia Government/Medical Research Council Joint Ethics Committee. All patients provided written informed consent for the collection of samples and subsequent analysis.

### The Gambian Tuberculosis Case Contact Study

In the TBCC study, we followed 317 adult sputum smear and culture positive tuberculosis index cases and 2348 of their household contacts. Participants were recruited between September 2002 and September 2004. Household members were eligible for inclusion in the study if they had been sleeping in the same compound (walled group of houses) as the index case during the index case's period of illness with TB. All contacts underwent a clinical assessment and had a Tuberculin Skin Test (TST: 2 tuberculin units (TU) of Purified Protein Derivative (PPD) RT23, Staten Serum Institute, Denmark) using the Mantoux technique. Subjects with skin test induration of ≥10 mm diameter were categorised as TST positive. Those with a negative TST at baseline had a repeat test after 3 months.

### Follow-up

Study participants were followed formally for 2 years and passively after that. Each individual was re-evaluated for symptoms of tuberculosis at each visit. All TB suspects received a chest radiograph and sputum analysis for acid fast bacilli (AFB) smear and culture. If tuberculosis disease was bacteriologically confirmed, patients were referred for the standard six month tuberculosis treatment course. Blood samples were taken at 3 months and 18 months following the initial visit and were processed to obtain Peripheral blood mononuclear cells (PBMC), plasma and RNA which were cryopreserved until required. Subjects were included if they were >18 years of age and were HIV-1 sero-negative. Ethical approval was obtained from the Gambia Government/Medical Research Council joint ethics committee.

### Study group definitions

All contacts with symptoms consistent with TB, that commenced at least 3 months after their respective index case was diagnosed, were considered to be possible secondary TB cases (termed progressor throughout this paper). A TB diagnosis was based on chest x-ray and positive sputum smear and culture results, and/or their response to TB treatment. Randomly chosen non-progressors were age and sex-matched to the progressors and were diagnosed as definitely not having TB for the whole follow-up period. Non-progressors were selected from different household as the progressors to reduce effects of clustering.

### PBMC thawing and flow cytometry

PBMC were removed from liquid nitrogen and semi-thawed in a 37°C water bath. They were then quickly resuspended in cold RPMI+10%FCS and centrifuged (1500 rpm, 5 min), followed by a second wash to remove residual DMSO. Cells were then resuspended in RPMI+10% FCS and counted. For flow cytometry staining, at least 200,000 cells were used per test. After carefully removing the supernatant, 20 µL of previously titrated antibody cocktail was added to each tube and vortexed. Cells were then incubated at 4°C for 15 min, followed by a wash with cold PBS/FCS/Azide buffer. Supernatant was removed and cells resuspended in 1% paraformaldehyde for flow cytometry acquisition. Antibodies used were CD4-PerCP, CD8-Pacific Blue, CD27-APC, CD45RO-PE, CD56-PE, CD56-PECy7 (all from BDPharmingen, USA); CD3-PE-Cy7, CD19-APCAlexa750 (all from eBioscience, UK) and Vα24-FITC and Vβ11-PE (Beckman Coulter, USA). All samples were acquired with a 9-colour (11-parameter) CyAn ADP™ flow cytometer (Beckman Coulter, USA). Prior to acquisition, calibration and compensation were performed and lymphocytes gated according to 90° forward and side scatter plots. FACS plots were analysed using FlowJo software (Treestar, OR), version 6.1.1.

### Multiplex cytokine analysis of plasma samples

Plasma samples from TB cases, non-progressors and progressors at recruitment, 3 months and 18 month time-points were analysed using a Bio-Rad custom made 7-plex kit according to the manufacturer's instructions. Cytokines assessed were: IL-10, IL-12(p40), IL-13, IL-17, IL-18, IFN-γ and TNF-α. Following pre-wetting of the filter plate, 50 µl of bead suspension was added to each well and washed twice. 50 µl of samples and standards were then added, the plate was sealed and shaken for 30 sec at 1100 rpm, and incubated for 1 hr at 300 rpm. The plate was washed 3 times then 25 µl of pre-diluted detection antibody was added. Following shaking, the plate was incubated for 30 min. at 300 rpm in the dark. After washing, 50 µl of 1× streptavidin-PE was added to each well and incubated for 10 min. The plate was again washed and resuspended in 125 µl of assay buffer, sealed, mixed and immediately read on the Bioplex analyser using Bioplex manager software (version 4.0; Bio-Rad, USA) and a low PMT setting. All standards were run in duplicate.

### Dual-colour Reverse Transcription Multiplex Ligation-dependent Probe Amplification (RT-MLPA)

RNA was isolated at MRC Unit, The Gambia from peripheral blood using Paxgene tubes and extraction kits according to the manufacturer's instructions (Qiagen) and shipped to Leiden University Medical Center (LUMC). Dual colour RT-MLPA was performed at LUMC [13], [14] with several major modifications [15], including probe-primer design for 45 genes of interest [15], [16]. Briefly, 100–150 ng RNA was reverse transcribed using gene-specific RT primers and MMLV reverse transcriptase. This was denatured and hybridized overnight at 60°C with a SALSA probe mix (MRC Holland, The Netherlands). After treating the samples with ligase-65 (MRC-Holland, The Netherlands) for 15 min at 54°C, PCR amplification was performed with specific SALSA FAM- or HEX-labelled MAPH primers (2 µM each, forward primer 5′-GGCCGCGGGAATTCGATT-3′ and reverse primer 5′-GCCGCGAATTCACTAGTG-3′), 13.75 µL H_2_0 and 0.25 µL SALSA polymerase [15]. PCR conditions were 33 cycles of 30 s at 95°C, 30 s at 58°C and 60 s at 72°C, followed by 1 cycle of 20 min at 72°C. PCR products were then diluted 1∶10 in HiDi formamide containing 400 HD ROX size standards and analysed on an ABI PRISM 3730 capillary sequencer (Applied Biosystems, UK). Data were analysed using GeneMapper software (Applied Biosystems, UK) and peak areas were exported to a Microsoft Excel file. Sample-related and peak-related differences in PCR and electrophoresis efficiency were corrected by adjusting to GAPDH housekeeping gene. Signals below the threshold value for noise cut-off (peak area ≤200) were adjusted accordingly. A positive control that encompassed the combined target-specific sequences of the left and right hand half-probes was used for all runs.

### Statistical Analysis

For Hematological, Immunophenotyping and Luminex analyses, group medians and distributions were compared using a Kruskal-Wallis test followed by Dunn's post-test comparison. For RT-MLPA analysis a Mann-Whitney U-test was performed. To avoid the assumption of constant variance within groups, robust variance estimates were used. Analyses were performed using STATA version 9.1 (Stata Corporation, USA) and Matlab version 7.6 (Mathworks, Natwick, 2008).

## Results

### Subject information

We analysed 22 confirmed TB progressors. These had PBMC and/or RNA and/or plasma samples available but not all sample types were available for all subjects at all time-points. Of the progressors, 14 had viable PBMCs stored at recruitment, 9 at 3 months and 9 at 18 months. These were matched with 31 non-progressors at recruitment, 35 non-progressors at 3 months and 22 non-progressors at 18 months. For cytokine levels within plasma samples, 13 progressors were analysed at each time-point and compared to 21 non-progressors. For RT-MLPA analysis, 12 progressors and 31 non-progressors were analysed. The median[IQR] age of the confirmed progressors was 25[20–45] with 58% males. The non-progressors were age and sex matched. To try and correct for the variation in the time taken to progress to active disease (median[IQR] of 507[187–714] days), we performed analyses based on early (progression between 90–507 days) versus late (progression >507 days) progressors where possible (indicated).

### Comparison of hematological parameters between progressors and non-progressors

**Figure 1:**
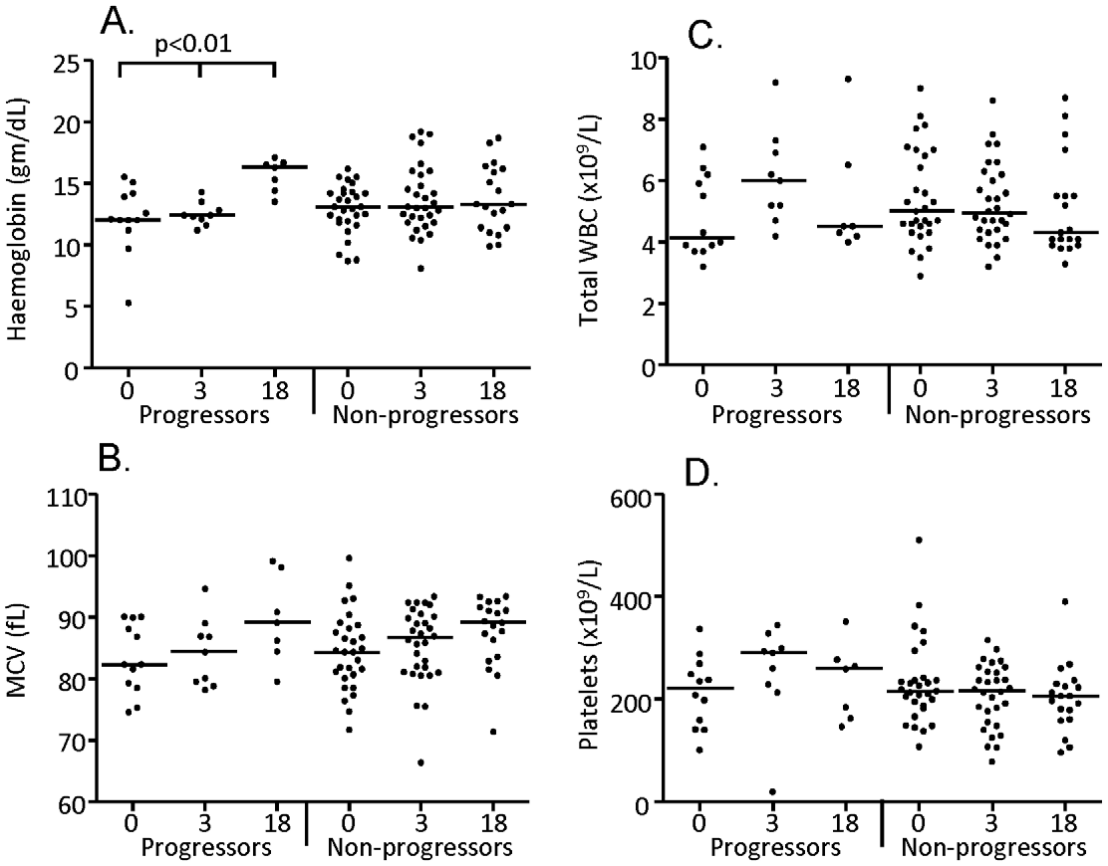
Analysis of hematological parameters in progressors versus non-progressors. Hemoglobin (A) and MCV (B) levels, Total white blood cell (WBC) (C), and platelet (D) counts, were obtained from the Hematology lab at MRC, Fajara. Bar indicates median of 13 progressors and 28 non-progressors at recruitment (0), 9 progressors and 30 non-progressors at 3-months (3) and 7 progressors and 19 non-progressors at 18 months (18). Data were analysed using a Kruskal-Wallis test followed by Dunn's post-test comparison. Significant differences are indicated.

There were no significant differences between progressors and non-progressors at any time-point for any of the hematological parameters analysed (WBC, Hemoglobin, MCV and platelets) (Fig. 1; Table 1). However, Hemoglobin levels were significantly increased at the 18 month time-point compared to recruitment for the progressors (median[IQR] = 15.3[14.4–16.7] and 12.1[11.6–14] respectively; p<0.01, Fig. 1A). When the progressors at recruitment were analysed based on those progressing early (<median time-point of 507 days; EP) compared to those progressing late (>median time-point of 507 days; LP), there was a significantly lower level of MCV in EP compared to LP (median[IQR] = 82[78–88] and 88[81–90] respectively; p = 0.043; Table 1).

**Table 1:** Hematological and Immunological peripheral blood analysis: comparison of non-progressors with total progressors, early progressors and late progressors at the recruitment time-point.

Subset	Early Progressors	Late Progressors	Total Progressors	Non-Progressors
**Hemoglobin (g/dL)**	13[11–14]	12[12–15]	12[12–14]	13[12–14]
**WBC (×10^9^/L)**	4.2[3.9–4.8]	6.1[3.9–6.6]	4.5[3.9–6.1]	5.1[4.4–7]
**MCV (fL)**	82[78–88]	88[81–90]	85[80–90]	84[80–89]
**Platelets (×10^9^/L)**	244[196–299]	217[141–286]	237[169–283]	215[184–237]
**% CD3**	52[38–61]	52[35–62]	52[35–60]	54[48–62]
**% CD4**	19[11–32]*	24[18–42]	21[17–34]*	30[22–37]
**% CD8**	17[10–31]	16[9–18]	16[10–22]	16[13–20]
**% B CELLS**	6.5[3–11]	8[6–10]	6.5[5–10]	11[8–16]
**% NK CELLS**	9.2[9–16]	13[8–21]	11.1[9–17]	12[6–19]
**% NKT CELLS**	0.08[0.05–0.13]*	0.06[0.02–0.09]	0.06[0.05–0.11]*	0.09[0.05–0.33]
**% γδ T CELLS**	7.3[3.8–14.6]	5.4[2.3–8.5]	7.3[2.5–11]	6.9[4.3–10.4]
**% CD4^+^CD25^+^**	1.3[0.7–1.8]	1.9[1.1–2.0]	1.5[1.0–1.9]*	1.1[0.7–1.4]
**% CD4^+^CD25^+^127^lo^**	0.6[0.4–1.1]	0.7[0.4–0.9]	0.6[0.4–0.9]	0.6[0.3–0.9]
**% CD4^+^IL7R^+^**	67[47–89]	80[78–91]	79[67–89]	80[72–88]
**%CD8^+^IL7R^+^**	47[26–54]	46[32–65]	46[32–59]	54[41–61]
**%CD4^+^ NAÏVE**	37[17–46]	39[27–59]	38[25–51]	35[21–43]
**%CD4^+^ CM**	32[29–43]	33[23–44]	32[27–40]	32[27–45]
**%CD4^+^ EM**	19[14–32]	16[11–29]	19[12–26]	18[14–27]
**%CD4^+^ TE**	10[8–14]	5[4–7]	7[5–10]	6[2.3–15]
**%CD8^+^ NAÏVE**	49[24–55]	45[10–53]	46[15–53]	43[25–55]
**%CD8^+^ CM**	15[9–18]	12[10–18]	13[10–18]	13[10–21]
**%CD8^+^ EM**	8[4–16]	11[6–43]	9[6–28]	10[4–16]
**%CD8^+^ TE**	33[25–48]	30[26–35]	31[26–38]	29[20–40]

Data expressed as median[interquartile range]. WBC = White Blood Cells; MCV = Mean Corpuscular Volume; Early Progressors (≤507 days to progression); Late Progressors (>507 days to progression); CM = central memory; EM = effector memory; TE = terminal effectors.

* = significantly different to non-progressors (please refer to text for actual p-values).

### Comparison of lymphocyte subsets between progressors and non-progressors

**Figure 2:**
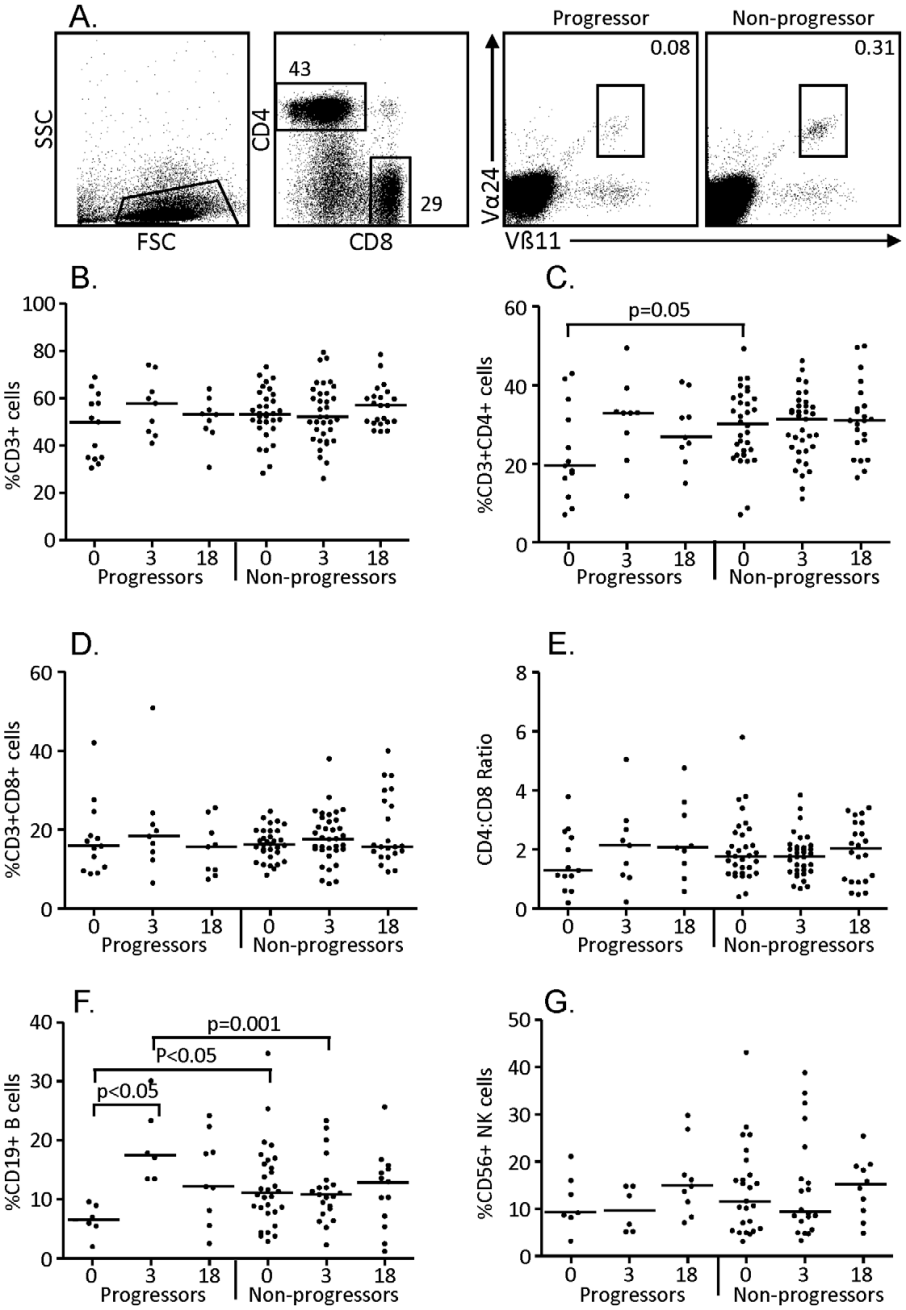
Percentage of T, B and NK cells in progressors and non-progressors. Cryopreserved PBMC were thawed and analysed. Representative FACS plots indicating our gating strategy are shown in (A): following gating on the lymphocyte population as determined by the FSC/SSC profile, we analysed lymphocyte populations. Shown are a representative CD4+ and CD8+ profile and a comparison of Vα24+Vβ11+ invariant NKT cell levels in a progressor (left) and non-progressor. All subjects were analysed for percentages of total T cells (B), CD4+ (C), CD8+ (D), CD4∶CD8 Ratio (E), B cells (F) and NK cells (G). Bars indicate median of 14 progressors and 31 non-progressors at recruitment (0), 9 progressors and 35 non-progressors at 3 months (3) and 9 progressors and 22 non-progressors at 18 months (18). Data were analysed using a Kruskal-Wallis test followed by Dunn's post-test comparison. Significant differences are indicated.

**Figure 3:**
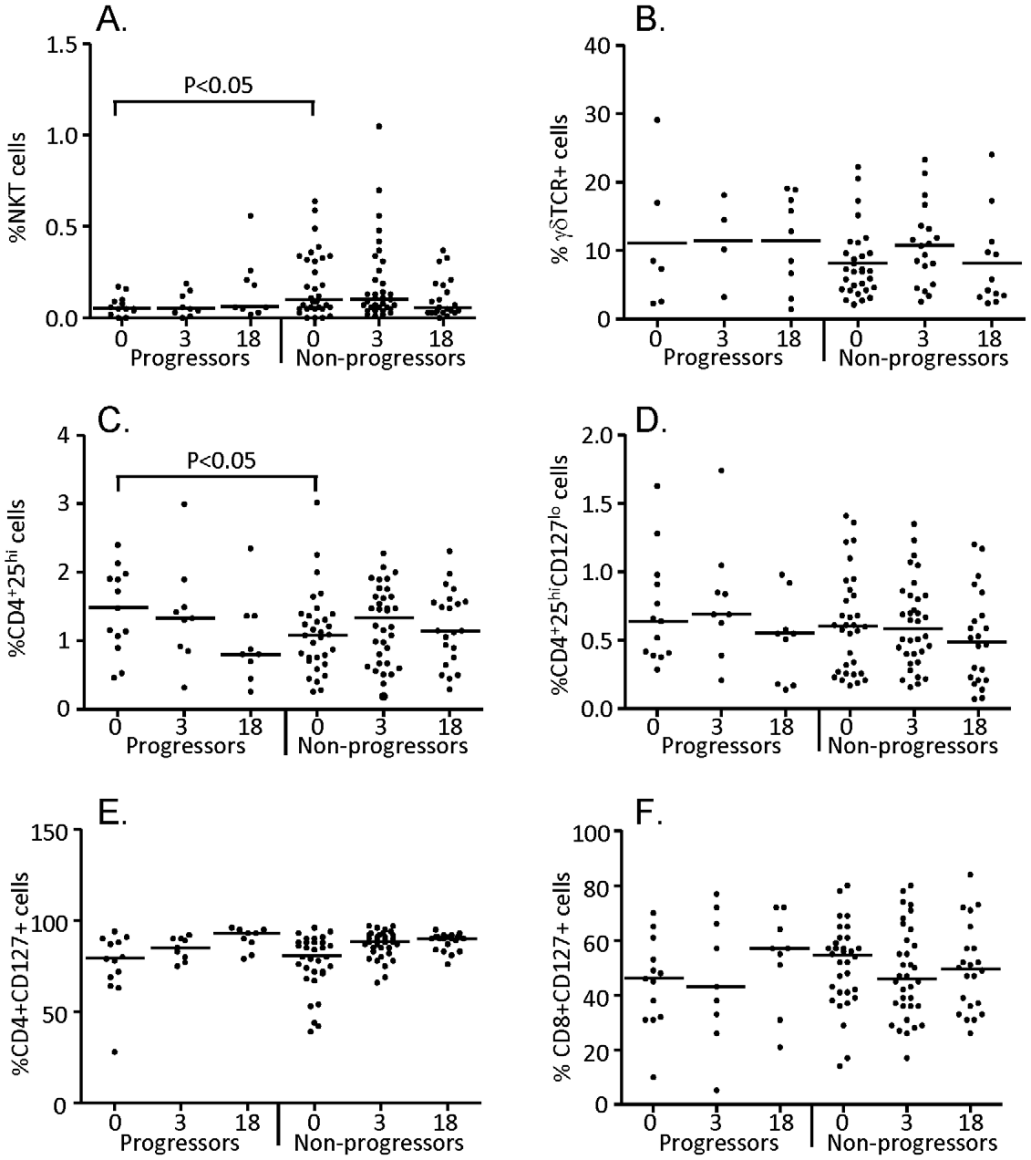
Percentage of NKT cells, γδ T cells, T regulatory cells and CD127 expression in progressors and non-progressors. Cryopreserved PBMC were thawed and analysed for percentages of Vα24+Vβ11+ NKT cells (A), γδ T cells (B), CD4+CD25^hi^ cells (C), CD4+CD25^hi^CD127^lo^ cells (D), CD4+CD127+ cells (E) and CD8+CD127+ cells (F). Bars indicate median of 14 progressors and 31 non-progressors at recruitment (0), 9 progressors and 35 non-progressors at 3 months (3) and 9 progressors and 22 non-progressors at 18 months (18). Data were analysed using a Kruskal-Wallis test followed by Dunn's post-test comparison. Significant differences are indicated.

Lymphocyte populations were gated according to FSC and SSC profile (Fig. 2A) and analysed for specific subsets (CD4+, CD8+ and NKT cell populations are shown (Fig. 2A). Analysis at recruitment showed a significantly lower level of CD4+ T cells from progressors compared to non-progressors (median[IQR] = 21[17–34] and 30[22–37] respectively; however this was not seen at the other time-points (Fig. 2C, Table 1). B cells were significantly higher at 3 months in progressors compared to non-progressors (p = 0.001; Fig. 2F, Table 1) but were relatively low at the recruitment time-point and were comparable to the non-progressors at 18 months. In progressors we also found a significantly lower frequency of Vα24+Vβ11+ NKT cells (median[IQR] = 0.06[0.05–0.11]; p<0.05) but a significantly higher level of CD4+CD25+ cells (median[IQR] = 1.5[1.0–1.9]; p<0.05) at recruitment compared to non-progressors (median[IQR] = 0.09[0.05–0.33] and 1.1[0.7–1.4] respectively; Figs. 3A and 3C; Table 1). However, no differences in T regulatory (CD4+CD25+CD127^lo^) cell frequency was observed, indicating that the CD4+CD25+ cells were most likely activated T cells. While we saw a trend towards a higher proportion of CD45RO+CD27+ (central memory) and lower levels of CD45RO−CD27− (terminal effector) cells in both the CD4+ and CD8+ subsets for progressors, these were not significantly different from non-progressors at any time-point (Table 1). However, we did see a significant decrease in the proportion of terminal effector cells within the CD4+ subset at 3 and 18 months compared to the recruitment time-point for the progressors (p<0.05 for both; data not shown). The only other subset that showed a difference between recruitment and 18 month time-points for the progressors were CD4+CD127+ cells (%) which were significantly higher at 18 months (p<0.01; Fig. 3F). We also compared early to late progressors at the recruitment time-point and found that CD4+ T cells were significantly lower in EP compared to LP (median[IQR] = 19[11–32] and 24[18–42] respectively; p = 0.029; Table 1) and also compared to the non-progressors (p = 0.002). Indeed, there were no significant differences between the LP and the non-progressors for any lymphocyte subset evaluated (Table 1). The lower proportion of NKT cells from progressors at the recruitment time-point was also due to a significant difference between non-progressors and the early progressors (median[IQR] = 0.09[0.05–0.33] and 0.08[0.05–0.13] respectively; p = 0.011; Table 1).

### Comparison of plasma cytokine levels in progressors and non-progressors

**Figure 4:**
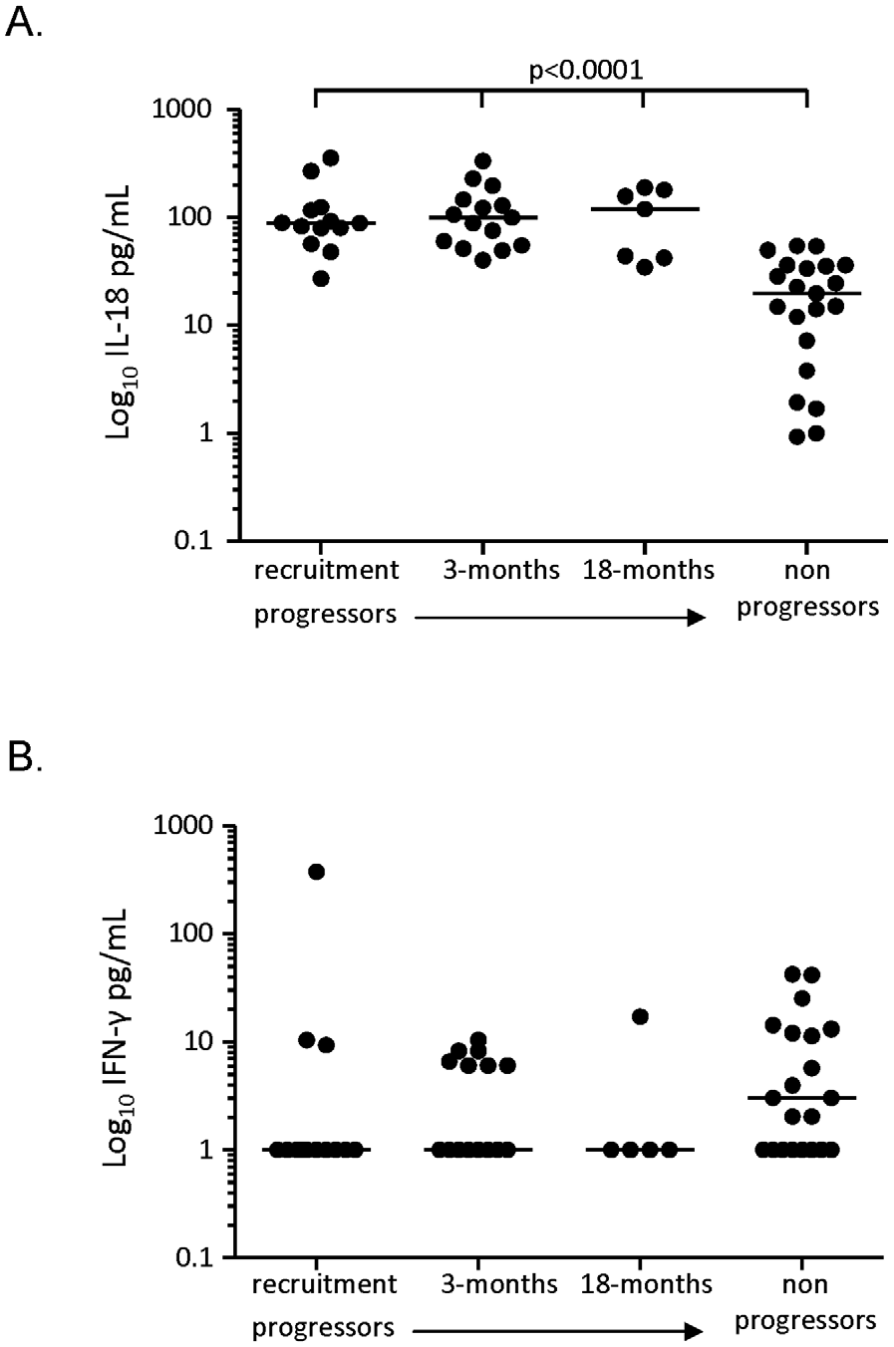
Analysis of cytokine levels in the plasma of progressors and non-progressors. 7-plex Luminex analysis was performed on cryopreserved plasma samples. Data shown are for IL-18 (A) and IFN-γ (B). Bars indicate median of 13 progressors at recruitment, 3 and 18 months and 31 non-progressors. Data were analysed using a Kruskal-Wallis test followed by Dunn's post-test comparison. Significant differences are indicated.

Analysis of ex-vivo plasma cytokine levels showed very low levels in all samples. IL-18 was detectable at the highest level for all groups and was significantly higher in progressors at all time-points compared to non-progressors (median[IQR] = 89[68–120], 99[55–147], 120[42–180] and 20[5–36] respectively; p<0.001 for all; Fig. 4A). No significant differences were observed for any other cytokine, presumably due to the low levels in all samples. However, the proportion of non-progressors with detectable levels of IFN-γ was higher than in the progressors at all time-points (Fig. 4B).

### Gene expression differences between progressors and non-progressors

We used RT-MLPA to detect differences in expression of specific genes between progressors and non-progressors. We saw a significantly lower level of Bcl2 in the progressors (median[IQR] = 2109[1616–2591] compared to non-progressors (2882[2123–1353] respectively; p = 0.011; Table 2). Conversely, progressors had significantly higher levels of chemokine receptor 7 (CCR7) compared to non-progressors (median[IQR] = 19126[17384–21655] and 15849[14602–19971] respectively). Analysis of the fold-differences between progressors and non-progressors showed the highest fold-difference (2.51) for Bactericidal Permeability Increasing gene (BPI) and the lowest for FCGR1A (0.57) in progressors compared to non-progressors, although neither of these were significant due to high standard deviations (Table 2). Levels of β2-microglobulin were similar between the groups (data not shown).

**Table 2:** Differences in RT-MLPA gene expression levels in progressors and non-progressors.

Description	Variable	Non-Progressors	Progressors	Fold difference
**apoptotic factors**	BCL2	2882[2123–3338]	2109[1616–2591]	0.73
	Caspase8	12034[10076–14078]	10960[8272–13771]	0.91
	TNFRSF1A	13682[11793–15626]	14396[10648–17683]	1.05
	TNFRSF1B	3656[3249–4180]	4124[3790–4620]	1.13
**Cytokines/chemokines**	TNF	200[200–1392]	200[200–1874]	1.00
	BLR1	1443[1035–1777]	1734[1273–2190]	1.20
	CXCL10	200[200–386]	271[200–746]	1.36
	CCL19	565[308–735]	537[200–650]	0.95
	CCL22	276[200–664]	375[200–695]	1.36
	CCL4	200[200–261]	200[200–200]	1.00
	CCR7	15849[14602–19971]	19307[17648–22192]	1.22
**Innate immunity**	CD163	1224[1144–1518]	1251[1154–1752]	1.02
	FPR1	14203[11336–19155]	13540[10369–20923]	0.95
	LTF	200[200–200]	200[200–200]	1.00
	**BPI**	**518[200–1677]**	**1300[281–2055]**	**2.51**
	NCAM1	1850[1532–2374]	1617[1345–2258]	0.87
**B cell factors**	CD19	597[249–946]	664[497–874]	1.11
	**FCGR1A**	**883[310–1270]**	**505[200–1811]**	**0.57**
	MMP9	200[200–539]	280[200–594]	1.40
	TIMP2	15285[12874–16946]	17429[13864–20035]	1.14
**T cell factors**	CD3e	21509[17293–25332]	21207[19301–22977]	0.99
	CD4	4165[3440–5119]	4880[3333–7075]	1.17
	CD8a	200[200–2560]	200[200–4699]	1.00
	IL7R	16796[13231–21510]	16048[13606–18574]	0.96
**Regulatory T cells**	CTLA4	200[200–436]	200[200–309]	1.00
	FOXP3	245[200–434]	235[200–475]	0.96
	TGFB1	5211[4616–5811]	5420[4579–6268]	1.04
	TGFBR2	5745[5114–6523]	6161[4583–7252]	1.07
**Intracellular trafficking**	RAB13	1073[396–1189]	1082[746–1384]	1.01
	RAB24	2229[1976–3125]	2678[2275–3096]	1.20
	Rab33A	200[200–287]	219[200–318]	1.10
	SEC14L1	15727[13077–19137]	18310[15395–26280]	1.16

Data expressed as median[interquartile range]. Fold difference is shown as expression in progressors compared to non-progressors. BPI showed the highest fold increase and FCGR1A the lowest.

## Discussion

This study provides the first detailed comparison of the immune system between contacts of TB cases who progress to active disease and those who don't progress, thus increasing our knowledge of what constitutes protective immunity in TB. The major differences between progressors and non-progressors included a significantly lower percentage of CD4+ T cells and NKT cells but significantly higher percentage of CD4+CD25+ cells in progressors at recruitment. We also found significantly higher plasma IL-18 levels and higher CCR7 but lower Bcl2 gene expression in progressors compared to non-progressors. These differences were generally due to early progressors: the immune system of non-progressors and late progressors was comparable at the recruitment time-point, whilst subjects who progressed between 90–507 days following recruitment showed the most immunological differences to non-progressors.

While we found significant differences at the hematological, lymphocyte, plasma and RNA levels, our analyses also raised many limitations which can only be addressed in a much larger cohort study such as the Gates Grand Challenge for TB [3]. Firstly, the number of progressors used in this study was small. While this could be overcome in part by increasing the number of non-progressor samples we analysed, it could not overcome the fact that we had such variability in progression time. Secondly, the interval between the 3 month and 18 month sample collection time points did not facilitate a precise analysis of the changes in the immune system prior to progression to active disease; which is highlighted by the fact that the early-progressors showed the most differences to the non-progressors. Future work should allow for increased time-points of sample collection to ensure the immune responses are captured at the closest possible time-point to progression.

Despite these difficulties, there were several parameters that showed differences between progressors and non-progressors and should be further validated as potential biomarkers for protective immunity to TB. Interestingly differences were observed in both the innate and the adaptive immune systems, reinforcing the requirements for triggering the innate immune response (through use of adjuvants) in the design of new and improved vaccines [17]. At the RNA level, we saw the highest fold-difference in BPI and the lowest fold-difference in FCGR1A (CD64) in progressors compared to non-progressors; both of which are expressed by neutrophils and are important in the control of bacterial infections [18], [19]. CD64 is important in the immune response to TB with defects in this gene resulting in increased susceptibility to TB [19], although recent work has shown subjects with active disease to have significantly higher levels of this gene in comparison to latently infected individuals [8]. IL-18 is produced by the innate immune system, acts as a precursor to IFN-γ production and is highly increased in patients with advanced tuberculosis [20], [21]. This suggests that the observed increase in plasma IL-18 levels in progressors compared to non-progressors is important in the early stages of the immune response to *Mycobacterium tuberculosis* infection. No difference in NK cell proportion was observed at any time-point but a significantly lower proportion of invariant NKT cells were seen in progressors compared to non-progressors. Invariant NKT cells act as a link between the innate and adaptive immune systems and are important in the control of bacterial infections [22], [23], thus a reduction in NKT cell numbers will invariably have downstream effects on the efficacy of the adaptive immune response. The major difference in the adaptive immune system was a significantly lower proportion of CD4+ T cells in progressors compared to non-progressors. The crucial role of CD4+ T cells in protection against TB disease progression is supported by the profound increase in TB associated with HIV-induced CD4 depletion [24]. The decrease in CD4+ T cells in progressors may be associated with the decreased levels of the anti-apoptotic gene, Bcl2 that we observed. The role of apoptosis in protection against TB disease is complicated as there are differential effects depending on the cell type involved. For instance, induction of apoptosis of infected macrophages is crucial for control of disease [25] and is induced by TNF-α [26]. It is thought this allows removal of infected cells while minimizing tissue destruction in adjacent, uninfected cells [27]. Conversely, apoptosis of T cells is detrimental to control of disease progression [25]. Thus, further studies should separate cells into monocytes and lymphocyte subsets in order to identify which cells are affected by the reduced Bcl2 expression. Indeed, a recent study which separated lymphocytes from monocytes prior to RT-MLPA, showed differential levels of Bcl2 in patients with sepsis compared to controls in the lymphocyte but not monocyte populations [28].

In conclusion, this study provides the basis for further exploration of protective immunity to TB using a case-contact study platform and nested case-control study. Our findings suggest that several markers may predict the onset of active TB in exposed asymptomatic household contacts. These markers, once validated in a larger cohort study, may help to prospectively identify patients at risk of developing active TB as well as demonstrating natural protective immune requirements for the next generation of TB vaccines. Once these markers have been validated in larger studies, they provide avenues to prospectively identify people at risk of developing TB, a key issue in the testing of new TB vaccines [29].
